# Role of glycosylation in TGF-β signaling and epithelial-to-mesenchymal transition in cancer

**DOI:** 10.1007/s13238-020-00741-7

**Published:** 2020-06-25

**Authors:** Jing Zhang, Peter ten Dijke, Manfred Wuhrer, Tao Zhang

**Affiliations:** 1 Oncode Institute and Cell Chemical Biology, Leiden University Medical Center, 2300 RC Leiden, The Netherlands; 2 Center for Proteomics and Metabolomics, Leiden University Medical Center, Leiden, The Netherlands

**Keywords:** cancer, epithelial-to-mesenchymal transition, glycosphingolipids, *N*-glycosylation, *O*-glycosylation, transforming growth factor-β

## Abstract

Glycosylation is a common posttranslational modification on membrane-associated and secreted proteins that is of pivotal importance for regulating cell functions. Aberrant glycosylation can lead to uncontrolled cell proliferation, cell-matrix interactions, migration and differentiation, and has been shown to be involved in cancer and other diseases. The epithelial-to-mesenchymal transition is a key step in the metastatic process by which cancer cells gain the ability to invade tissues and extravasate into the bloodstream. This cellular transformation process, which is associated by morphological change, loss of epithelial traits and gain of mesenchymal markers, is triggered by the secreted cytokine transforming growth factor-β (TGF-β). TGF-β bioactivity is carefully regulated, and its effects on cells are mediated by its receptors on the cell surface. In this review, we first provide a brief overview of major types of glycans, namely, *N*-glycans, *O*-glycans, glycosphingolipids and glycosaminoglycans that are involved in cancer progression. Thereafter, we summarize studies on how the glycosylation of TGF-β signaling components regulates TGF-β secretion, bioavailability and TGF-β receptor function. Then, we review glycosylation changes associated with TGF-β-induced epithelial-to-mesenchymal transition in cancer. Identifying and understanding the mechanisms by which glycosylation affects TGF-β signaling and downstream biological responses will facilitate the identification of glycans as biomarkers and enable novel therapeutic approaches.

## Introduction

Glycans are part of glycoproteins, proteoglycans, glycosaminoglycans (GAGs) and glycolipids which cover the cell surface. They play key roles in different biological and cellular functions. Protein glycosylation includes *N*-linked glycosylation (in which glycan is attached to a nitrogen of an asparagine (Asn) residue of a protein), *O*-linked glycosylation (in which glycans are attached to a serine (Ser) or threonine (Thr) residue of a protein), *C*-mannosylation (in which a mannose is attached to a Tryptophan (Trp) of a protein), phospho-glycosylation and glypiation (Krasnova and Wong, 2016; Reily et al., 2019). When proteins are heavily glycosylated and contain a core protein with one or more GAG chain(s) covalently attached via xylose(s), they are named proteoglycans (Iozzo and Schaefer, 2015). Glycolipids are carbohydrate-modified lipids, and this type of glycoconjugate includes glycosphingolipids (GSLs) (D’Angelo et al., 2013). Perturbed glycosylation has been linked to many developmental disorders, diseases and tumor progression (Pinho and Reis, 2015; Rodrigues et al., 2018). Many glycans on the surface of cancer cells have recently been identified as critical regulators controlling several pathological processes during tumor progression (Dube and Bertozzi, 2005; Freire-de-Lima, 2014).

Alterations in protein- and lipid-linked glycans are associated with a multitude of biological processes related to cancer. Because of their special cell-surface position, glycans are of critical importance in controlling cell-cell communication, signal transduction and receptor activation. Various glycan structures have already been characterized as hallmarks of cancer which allow cancer to survive, proliferate, become migratory and invasive (Wang et al., 2019). Currently, glycoproteins are the most used cancer biomarkers in the clinic, such as alpha-fetoprotein (AFP) for hepatocellular carcinoma (Leerapun et al., 2007; Cheng et al., 2014), cancer antigen 125 (CA125) for ovarian cancer (Dochez et al., 2019), carcinoembryonic antigen (CEA) for colon cancer (Auclin et al., 2018), and prostate specific antigen (PSA) for prostate cancer (Albertsen, 2018). In addition, glycan-related carbohydrate antigen 19-9 (CA19-9), also known as sialyl-Lewis A, is a key hallmark used routinely in the management of pancreatic ductal adenocarcinoma (PDAC) (O’Brien et al., 2015). It has a 79%–81% sensitivity and 82%–90% specificity for diagnosis of pancreatic cancer in symptomatic patients (Ballehaninna and Chamberlain, 2012). Proteoglycans especially glypican-1 (GPC1), which enriched on cancer-cell-derived exosomes, may play a role as a biomarker to detect early stages of pancreatic cancer (Melo et al., 2015).

Tumor initiation and progression mediated by (epi)genetic changes result in altered gene functions, including gain-of-function modifications in proto-oncogenes and loss-of-function modifications in tumor suppressor genes (Hanahan and Weinberg, 2000). Whereas growth factors, such as platelet-derived growth factor (PDGF) and epidermal growth factor (EGF), become overly active, the cytostatic action of growth inhibitory factors, such as transforming growth factor-β (TGF-β), is lost or corrupted (Heldin, 2004). These changes impact the cancer cell phenotype, which may be associated with increased proliferation, migration and invasion and/or creation of a favorable tumor microenvironment that drives angiogenesis, metastasis and/or immune evasion (Hanahan and Weinberg, 2011). The epithelial-to-mesenchymal transition (EMT) is an important step in cancer cell invasion and migration and is characterized by a change in cell morphology from a cobble stone epithelial-type shape to an elongated spindle-shaped fibroblast-like appearance (Derynck and Weinberg, 2019; Lu and Kang, 2019). The multifunctional cytokine TGF-β is known to be a crucial driver of EMT in various (cancer) cells (Derynck et al., 2014; Hao et al., 2019). TGF-β transduces signals via a single-pass transmembrane Ser/Thr kinase receptors and co-receptors, which have glycosylated extracellular domains (Heldin and Moustakas, 2016). Extracellular (and intracellular) signaling through TGF-β is intricately regulated, involving the glycosylation of cell surface TGF-β-binding proteins. These changes in the glycosylation are of critical importance for the cellular responses induced by TGF-β, including the EMT.

In this review, we first provide a general overview of glycosylation modifications and their roles in cancer. Next, we discuss advances in the understanding of how the glycosylation of TGF-β-signaling components affects their function. Thereafter, we review the changes in glycosylation in response to TGF-β that have been documented and focus in particular on those that are involved in TGF-β-induced EMT. Furthermore, we conclude by offering perspectives on how insights into the interplay between glycosylation and TGF-β signaling can be used for future diagnostic and therapeutic gains for cancer patients.

## Glycoconjugates and glycosylation

**Figure 1:**
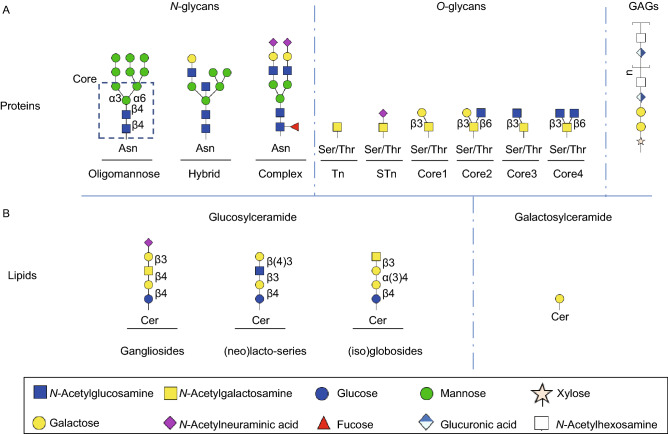
**Major classes of glycans in mammalian cells**. (A) *N*-glycans are linked to asparagine (Asn) residues of proteins and contains three different types which are oligomannose, hybrid and complex structures. These three *N*-glycans share a common core structure (indicated in dashed box). Mucin-type *O*-glycans are attached to a subset of serines (Ser) or threonines (Thr) and start with a single *N*-acetylgalactosamine (also known as Tn-antigen) then is extended by galactose or sialic acids or GlcNAc with four different cores. In addition, the O-xylose linked, non-branched glycosaminoglycans (GAG) are a large glycan family. (B) Glycosphigolipids (GSLs) include two precursor groups, galactosylceramides and glucosylceramides. The latter group contains three core structures: gangliosides, (iso)globosides, and (neo)lacto-series GSLs.

The biosynthesis of diverse glycan structures is based on the tight regulation and dynamic action of different enzymes, such as glycosyltransferases and glycosidases (Xu et al., 2018). Glycoproteins may carry *N*-linked glycans covalently attached to the nitrogen on the side chain of an asparagine residue. *N*-glycans contain a common pentasaccharide core region consisting of Manα1,6 (Manα1,3) Manβ1,4GlcNAcβ1,4GlcNAcβ1-Asn (Man_3_-GlcNAc_2_Asn) (Fig. 1A). They can be elaborated further, resulting in three main *N*-glycan types: oligomannosidic, hybrid and complex-type structures (Fig. 1A). *O*-linked glycans (*O*-glycans) are attached to a side chain at serine or threonine residues. *O*-linked α-*N*-acetylgalactosamine (*O*-GalNAc) or mucin-type *O*-glycan is a common type of *O*-glycan initiated via a single *N*-acetylgalactosamine residue that is attached to a Ser/Thr residue of a protein by glycosyltransferases (GTs) (Fig. 1A) (Brockhausen and Stanley, 2015). Once this initial structure is formed, additional sugars can be added. There are other types of *O*-glycans, such as *O*-linked *N*-acetylglucosamine (*O*-GlcNAc) or those attached to proteins via *O*-mannose, *O*-galactose, *O*-fucose or *O*-glucose (Ma and Hart, 2014; Haltiwanger et al., 2015; Pinho and Reis, 2015).

GSLs are the most common glycolipids in vertebrates and are composed of a carbohydrate moiety linked to a ceramide. GSLs can be grouped along two precursor groups, galactosylceramides (GalCer) and glucosylceramides (GlcCer), depending on the initial monosaccharide, which is attached via a β-glycoside bond to a ceramide molecule (D’Angelo et al., 2013). The latter group consists of three major series based on the synthesis pathways and core structures: gangliosides, (iso)globosides, and (neo)lacto-series GSLs (Fig. 1B) (Schnaar and Kinoshita, 2015). Many cell surface proteins are associated with GSLs, resulting in important roles for GSLs in regulating cell proliferation (Regina Todeschini and Hakomori, 2008), differentiation (Breimer et al., 2017) and tumor progression (Furukawa et al., 2019).

Proteoglycans (PGs) are a ubiquitous family of glycoconjugates composed of a core protein and one or several covalently attached GAG chains (Iozzo and Schaefer, 2015). GAGs are a family of highly sulfated and linear polysaccharides with repeating disaccharide unites (Fig. 1A). Based on the difference of repeating unites, GAGs are further divided into four groups: hyaluronan, chondroitin sulfate, heparan sulfate and keratan sulfate (Lindahl et al., 2015). Different forms of proteoglycans are present in nearly all extracellular matrices of connective tissues and are involved in regulating collagen fibril formation and the activity of secreted factors involved in communication between cells, including TGF-β.

## Glycosylation alterations in cancer

Many glycoconjugates, such as glycoproteins and glycolipids, are found on the outer surface of the cellular membrane. Because of this special position, glycans play essential roles in recognizing the extracellular matrix, interacting with other cells in the cellular microenvironment, regulating the binding of canonical protein ligands to their specific receptors and resulting in changes in cell-cell adhesion and signal transduction (Fuster and Esko, 2005; Pinho and Reis, 2015; Rodrigues et al., 2018). Changes in glycosylation of lipids and cell surface proteins have been shown to be associated with defects in basic biological processes observed in cancer, such as cell-cell adhesion (Zhao et al., 2008; Pinho et al., 2009; Pinho et al., 2013), cell-matrix interaction (Zhao et al., 2008), intercellular and intracellular signaling (Boscher et al., 2011; Gomes et al., 2013; Takeuchi and Haltiwanger, 2014), and cellular metabolism (Dennis et al., 2009; Bassaganas et al., 2014). In the remaining part of this section, we provide a few examples for illustration.

Epithelial cadherin (E-cadherin) is a cell-cell adhesion molecule, and its dysfunction or inactivation can contribute to cancer progression (Mendonsa et al., 2018). E-cadherin can be modified with β1,6-*N*-acetylglucosamine (β1,6GlcNAc)-branched structures, which are catalyzed by *N*-acetylglucosaminyltransferase V (*MGAT5*) and then become destabilized (Taniguchi and Kizuka, 2015). The disorganization of E-cadherin/catenin complex formation can result in an impaired cell-cell aggregation and epithelial cells acquiring an invasive phenotype (Pinho et al., 2013).

Integrins, as transmembrane receptors, are involved in extracellular matrix (ECM)–cell and cell–cell interactions as well as signal transduction (Marsico et al., 2018). Aberrant *O*-glycosylation on integrins can mediate the invasive phenotypes of hepatocellular carcinoma (HCC) tumor cells. Modification of integrin β1 by core 1 β1,3-galactosyltransferase (*C1GALT1*) regulates integrin activity, and overexpression of *C1GALT1* results in increased T antigen and sialyl T antigen levels and induces HCC cell migration and invasion (Liu et al., 2014a; Liu et al., 2014b). Core fucosylation is essential for the function of integrin and integrin-mediated cell migration and signal transduction in embryonic fibroblasts (Zhao et al., 2006).

Cell surface glycans can promote or hinder the cellular receipt of signals from outside by regulating the glycosylation of signaling specific receptors on the surface (Ferreira et al., 2018). Numerous key growth factors, such as EGF, hepatocyte growth factor (HGF), vascular endothelial growth factor (VEGF) and TGF-β (the focus of this review, see below), are involved in regulating tumor growth, invasion and metastasis (Lau et al., 2007). Altered glycosylation of the receptors for these growth factors can modulate their turnover, interaction with ligands and recruitment of other signaling proteins (Ferreira et al., 2018). For example, the *N*-glycan core fucosylation of EGFR is essential to regulate the EGFR-mediated intracellular signaling pathway. Knocking down fucosyltransferase 8 (*FUT 8*) blocked the phosphorylation of EGFR, decreased EGF-mediated signal transduction and inhibited EGF-mediated cellular growth. It has been proposed that the fucosylation of EGFR may promote its binding affinity for EGF or increase the propensity of EGFR to form dimers (Matsumoto et al., 2008). Moreover, the enrichment of gangliosides in the cell membrane has been shown to play a role in decreasing the phosphorylation of VEGFR2 and suppressing tumor angiogenesis in human endothelial cells (Mukherjee et al., 2008). Thus, studying glycosylation changes and unravelling how glycans modulate cellular signaling involved in cancer progression are of great importance and may potentially contribute to the development of novel therapeutic approaches.

## TGF-β signaling pathway

**Figure 2:**
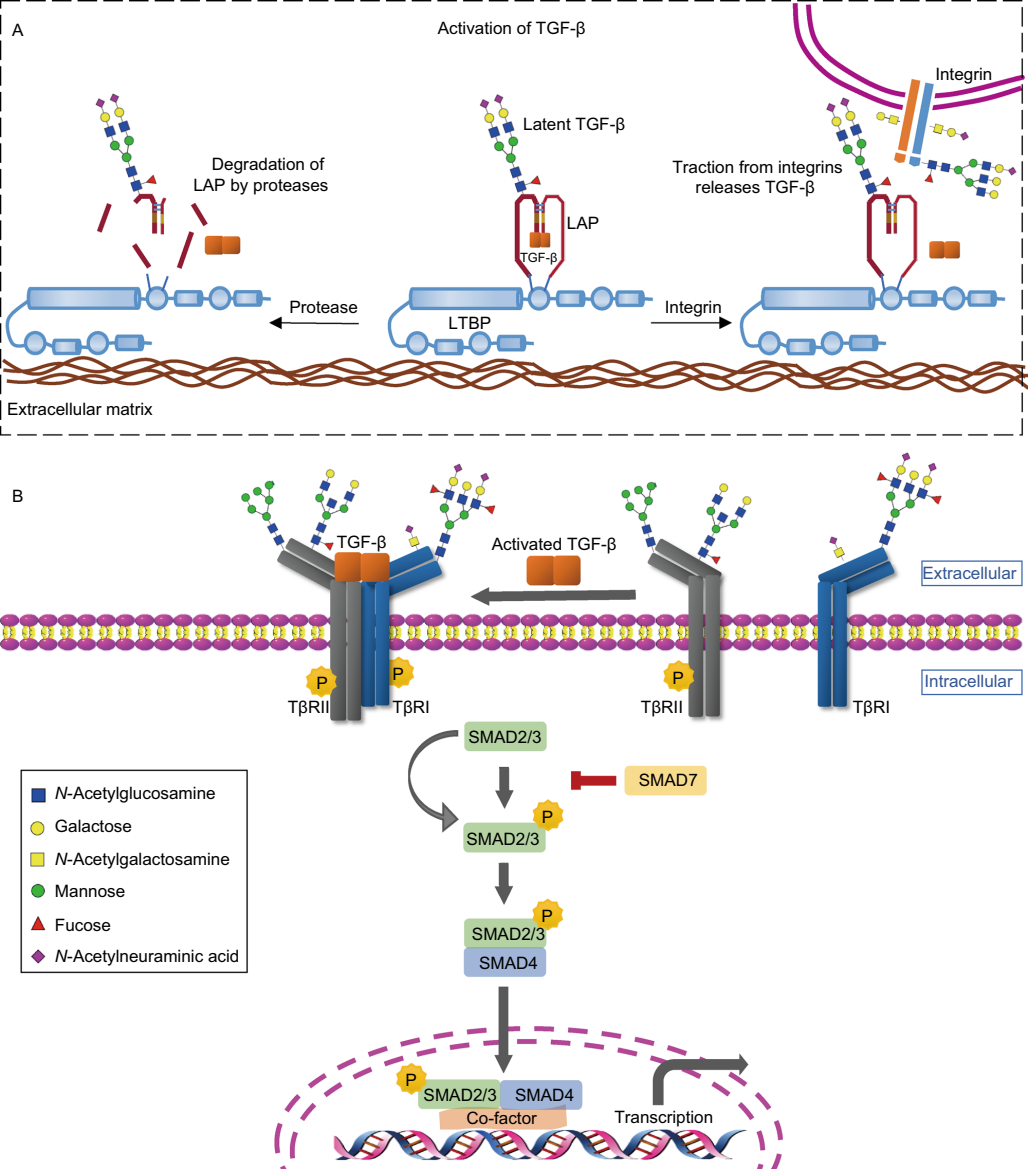
**Glycosylation changes in TGF-β activation and SMAD-dependent pathway**. (A) Activation of TGF-β. The mature TGF-β is noncovalently bound to the latency-associated peptide (LAP) and forms a latent TGF-β complex with the latent TGF-β-binding protein (LTBP). TGF-β can be released from the latent complex via cleavage of LAP by proteases digestion or integrin-dependent activation. The secreted TGF-β precursor contains N-linked complex type structures. (B) Canonical SMAD-dependent pathway. Receptor signaling starts with active TGF-β binding to the TGF-β type II receptor (TβRII), a constitutively activated kinase, which phosphorylates the TGF-β type I (TβRI), both located in the plasma membrane. Then the actived TβRII/TβRI complex phosphorylates the SMAD2/3, which can form heteromeric complexes with SMAD4. These complexes translocate into nucleus where they can modulate the transcription of target genes. Both TβRII and TβRI can be *N*- and *O*-glycosylated. Oligomannosidic, branching structures and core fucosylation are important for the localization and function of receptors. In addition, Lewis antigens attached on TβRI are observed in cancer cells

This review focuses on TGF-β, which is one of the key soluble factors in intercellular (mis)communication in cancer (Colak and Ten Dijke, 2017; Batlle and Massague, 2019). Three distinct isoforms have been identified, i.e., TGF-β1, TGF -β2 and TGF -β3. Here, we use TGF-β, unless a specific property has been shown for a specific isoform, in which case the isoform will be indicated. TGF-β is secreted by cells as part of an inactive biological complex, in which the mature carboxy-terminal TGF-β is noncovalently bound to its amino-terminal precursor fragment, also known as the latency-associated peptide (LAP) (Robertson and Rifkin, 2016). This small latent TGF-β complex can be covalently associated with the latent TGF-β-binding protein (LTBP); together, they compose the large latent TGF-β complex (Robertson et al., 2015). The LTBP facilitates the secretion of TGF-β and plays a role in targeting TGF-β to particular extracellular stores by interacting with the extracellular matrix. Latent TGF-β can be released via the action of specific proteases that cleave LAP or by mechanical forces in an integrin-dependent process (Fig. 2A) (Hyytiainen et al., 2004; Dong et al., 2014). Active TGF-β is capable of binding to receptors with intrinsic serine/threonine kinase activity, i.e., TGF-β type I (TβRI) and TGF-β type II (TβRII) receptors (Massague, 2000). TGF-β initially binds with TβRII, and thereafter, TβRI is recruited, forming a heteromeric complex (Fig. 2B). Subsequently, the TβRII kinase transphosphorylates the serine and threonine residues in the glycine-serine-rich (GS) juxtamembrane domain of TβRI (Heldin et al., 1997). This phosphorylation leads to the activation of the TβRI kinase and initiation of intracellular signaling. Intracellular TGF-β signaling is largely mediated by the Sma and Mad related (SMAD) family of proteins. The activated TβRI/TβRII complex phosphorylates the two C-terminal serine residues of receptor-specific SMADs (R-SMADs), i.e., SMAD2 and SMAD3. Then, activated SMAD2/3 can form a complex with a common SMAD mediator, i.e., SMAD4, and translocate into the nucleus where the heteromeric complex modulates the transcription of target genes (Budi et al., 2017). In addition, posttranslational regulation of the receptors and SMADs help define their stability and functions, thus provide negative feedback mechanisms of TGF-β/SMAD signaling (Xu et al., 2012a). Therefore, by signaling through the canonical SMAD-dependent pathway, TGF-β exerts its physiological and pathological actions through the transcriptional and posttranscriptional modulation of gene expression (Fig. 2B) (Hill, 2016). In addition to canonical SMAD-dependent signaling, SMAD-independent pathways can also be activated directly by ligand-occupied receptors to modulate downstream cellular responses in specific cell types (Zhang, 2017). Every step of the TGF-β pathway is precisely controlled at the extracellular and intracellular levels, and the components engage in cross talk with factors in other pathways (Massague, 2012; Luo, 2017). Cell surface co-receptors such as endoglin and betaglycan (also termed CD105 and TβRIII, respectively) play important roles in controlling the intensity, duration, specificity and diversity of signaling. Co-receptors are different from TβRI and TβRII in that they have larger extracellular domains but lack a functional enzymatic signaling motif (Nickel et al., 2018). Their domains contain a limited number of motifs, such as GAG modifications and the zona pellucida (ZP-1) domain (Kirkbride et al., 2005). It has been demonstrated that endoglin forms a complex with betaglycan and interacts with TGF-β family ligands and/or type I and type II receptors (Nickel et al., 2018).

## TGF-β-induced EMT in cancer progression

At the primary tumor site, the induction of the EMT program allows cells to acquire an invasive phenotype and drive cancer progression (Derynck and Weinberg, 2019; Lu and Kang, 2019). The EMT is a reversible process in which epithelial cell–cell contacts and apical–basal polarity are lost/decreased and in which cells acquire a mesenchymal phenotype with enhanced motility and invasion ability. The mesenchymal phenotype is apparent from the increased expression of cytoskeletal proteins, such as vimentin, and the upregulation of extracellular matrix proteins, such as collagens and fibronectin. In addition, the expression of epithelial markers, such as E-cadherin and Zona occludens protein (ZO-1), is downregulated concomitantly with an increase in the expression of mesenchymal marker proteins, including N-cadherin (Katsuno et al., 2013; Moustakas and Heldin, 2016). However, the transition from an epithelial to a mesenchymal state is often incomplete and results in intermediate states that retain both epithelial and mesenchymal characteristics. Recently, new guidelines and definitions for epithelial to mesenchymal transition recommended to use the term of epithelial–mesenchymal plasticity (EMP) to describe the cells undergoing intermediate E/M phenotypic states (Yang et al., 2020). This plasticity refers to as partial EMT, hybrid E/M status, a metastable EMT state, EMT continuum and EMT spectrum (Yang et al., 2020). TGF-β acts as a potent inducer of cancer progression by driving the EMT in both SMAD and non-SMAD signaling pathways. The TGF-β-SMAD signaling pathway directly activates the expression of EMT transcription factors, including the zinc finger transcription factors SNAIL and SLUG, two-handled zinc finger factors ZEB (zinc finger E-box-binding homeobox) 1 and ZEB2, and the basic helix-loop-helix factor TWIST (Katsuno et al., 2013; Moustakas and Heldin, 2016). TGF-β-induced non-SMAD pathways, such as the p38 MAPK (Yu et al., 2002) and PI3K/AKT/mTOR (Lamouille et al., 2012) pathways, also contribute to TGF-β-induced EMT.

## Glycan Modulation of TGF-β Signaling Components

### Effect of glycosylation on TGF-β secretion and bioavailability

Glycosylation of multiple proteins and complexes in the TGF-β signaling pathway regulates TGF-β secretion and bioavailability. LAP, which is noncovalently associated with TGF-β in an inactive complex, is glycosylated (Table 1) (Yang et al., 1997). β1-LAP contains three *N*-glycosylation sites at residues 82, 136, and 176 (Purchio et al., 1988). In the Chinese hamster ovary cell line, inhibition of *N*-glycosylation with either tunicamycin or an inhibitor of mannosidase II blocked the secretion of TGF-β1 (Fig. 2A) (Sha et al., 1989; McMahon et al., 1996). In human embryonic kidney cells, a mutation at the second *N*-glycosylation site of β1-LAP led to the blocked secretion of mature TGF-β1 and the inhibition of TGF-β1 bioactivity (Brunner et al., 1992; Lopez et al., 1992). The complex-type *N*-glycans present on secreted TGF-β1 precursor have been implicated in the maintenance of the latent complex (Fig. 2A) as removal of complex oligosaccharides containing sialic acid from LAP resulted in the dissociation of the TGF-β precursor from the latent complex (Miyazono and Heldin, 1989; Miyazono et al., 1992). In addition to LAP, LTBP has several potential *N*-glycosylation sites (Robertson and Rifkin, 2016), but whether the glycosylation of LTBP affects TGF-β release is still unclear.

**Table 1:** Glycosylation of TGF-β signaling components

Regulated signaling components		Glycan motif	Glycan type	Enzyme activity	Gene name	References
TGF-β secretion complexes	LAP	Oligomannosidic	*N*-linked			(Miyazono and Heldin, 1989; Miyazono et al., 1992)
		Complex structure				
	LTBP	*N*-glycans	*N*-linked			(Hubmacher and Reinhardt, 2009)
TGF-β receptors	TβRI/II	Oligomannosidic	*N*-linked			(Kim et al., 2012)
		Core fucose	*N*-linked	α1,6 fucosyltransferase 8	*FUT8*	(Wang et al., 2005; Lin et al., 2011)
		β1,6 branch	*N*-linked	*N*-acetylglucosaminyltransferase V	*MGAT5*	(Partridge et al., 2004)
		GM3	glycolipid	α2,3 sialytransferase 5, GM3 synthase	*ST3GAL5*	(Kim et al., 2013)
	TβRII	Sialylation	Both *N*- and *O*- linked			(Lee et al., 2013; Lee et al., 2015)
	TβRI	Sialyl-Lewis^A^	Both *N*- and *O*- linked	α1,4 fucosyltransferase 3	*FUT3*	(Hirakawa et al., 2014)
		Sialyl-Lewis^X^	Both *N*- and *O*- linked	α1,4 fucosyltransferase 3, 6	*FUT3*, *FUT6*	(Hirakawa et al., 2014)
		Lewis^Y^	Both *N*- and *O*- linked	α1,2fucosyltransferase 4	*FUT4*	(Li et al., 2010)
TGF-β Co-receptors	betaglycan	Heparin/chondroitin sulfate	HS/CS GAG			(Lopez-Casillas et al., 1991; Jenkins et al., 2018)
	Endoglin	*N*-glycans	*N*-linked			(Lux et al., 2000; Meurer et al., 2019)
	Neuropilin	*N*-glycans	*N*-linked			(Pellet-Many et al., 2008; Wu et al., 2017)
	SMAD2	*O*-glycans	*O*-linked			(Gotoh et al., 2020)

### Effect of glycosylation on TGF-β receptor function

**Figure 3:**
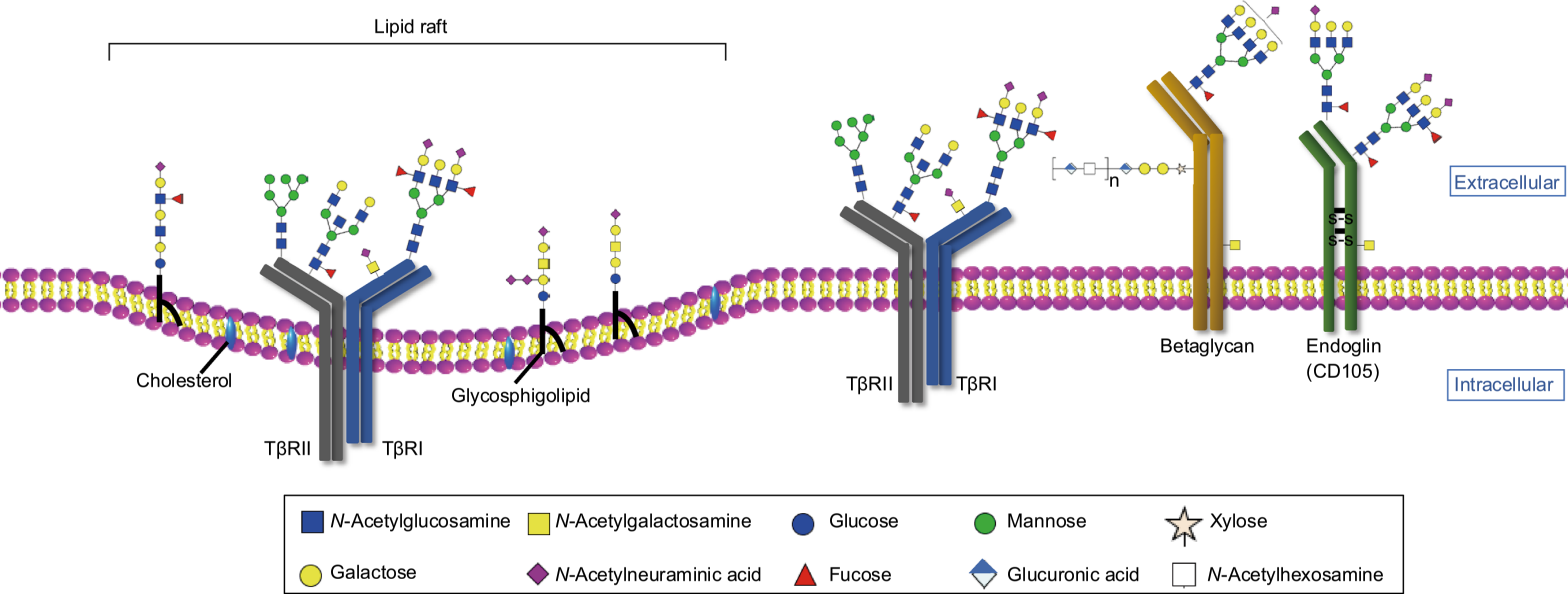
**Glycosylation of TGF-β receptors and co-receptors**. TGF-β receptors and co-receptors can be highly glycosylated with *N*-linked and *O*-linked glycans. Core fucosylation of TβRII and TβRI are required for their successful localization at the cell surface . In addition, the β1,6 branching structures of TβRII reinforces TGF-β signaling by inhibiting the endocytosis of TGF-β receptors. Lewis^X^ (sLe^X^) and sialyl-Lewis^A^ (sLe^A^) modified on TβRI are necessary for its activation. Betaglycan is composed of a core protein with covalently linked glycosaminoglycans (GAG) chains. Glycosphingolipids (GSLs), together with cholesterol, form microdomains, which are referred to as lipid rafts. The GSLs in these microdomains might paly a role in membrane trafficking of TGF-β receptors and signal transduction.

Glycosylation affects the TβRII localization in cells and interaction with TGF-β. Inhibiting or blocking the *N*-linked glycosylation of TβRII using glycosylation inhibitors including tunicamycin and kifunensine or by mutating *N*-glycosylation sites prevents TβRII proteins from being efficiently transported to the cell surface, resulting in decreased cellular sensitivity to TGF-β (Kim et al., 2012) (Table 1). Additional evidence shows that both complex type and a oligomannosidic type modification of TβRII are required for the successful cell surface transportation of TβRII (Kim et al., 2012). Core fucosylation of TβRII and TβRI has been studied as a key player in optimal TGF-β-receptor interactions and R-SMAD phosphorylation (Fig. 3, Table 1) (Venkatachalam and Weinberg, 2013). The TGF-β-induced phosphorylation of the SMAD2/3 proteins decreased when human renal proximal tubular epithelial cells were depleted of *FUT8*, a fucosyltransferase that specifically catalyzes core fucosylation of *N*-glycans (Lin et al., 2011). The data from Wang et al. (2005) also showed that lack of core fucosylation of TβRII results in the development of an emphysema-like phenotype in lung tissue. Mice deficient in *Fut8* exhibited a significantly high level of matrix metalloproteinase (MMP) expression, which is consistent with a deficiency in TGF-β1 signaling caused by dysregulation of TβRII. In contrast, upregulated expression of *FUT8* in mice resulted in high levels of core fucosylation of TGF-β type I and type II receptors, facilitating TGF-β binding and promoting downstream TGF-β signaling in breast cancer cells (Tu et al., 2017). The activation of these receptors further promoted cell migration and invasion. Branching of *N*-glycans catalyzed by *MGAT5* has been studied to promote galectin-3 expression on the cell surface and sensitivity of TGF-β signaling (Fig. 3) (Partridge et al., 2004). Elongation of a poly-*N*-acetyllactosamine chain on β1-6GlcNAc branches via *MGAT5* leads to the formation of a poly-*N*-acetyllactosamine structure (Nagae et al., 2018). This specific glycan structure is preferentially recognized by galectin-3, forming complexes between galectin-3 and *MGAT5*-modified *N*-glycans (Partridge et al., 2004; Priglinger et al., 2016). Depletion of *Mgat5* in mouse hepatic stellate cells downregulated expression of galectin-3 and inhibited the sensitivity of TGF-β1 to TGF-β receptors. Treatment of *Mgat5* knock down cells with nystatin, which is a chemical endocytosis inhibitor, promoted receptor accumulation in the membrane and rescued the sensitivity to TGF-β1. This provided further evidence that galectin-3 could form a lattice which reinforces TGF-β signaling by inhibiting the endocytosis of TGF-β receptors (Partridge et al., 2004).

In addition, sialylation has been shown to be associated with TβRII inactivation in colorectal cancer (CRC) cells. Altered sialylation and microsatellite instability (MSI) is a common feature of many malignancies, including CRC (Lee et al., 2013). The MSI phenotype is related to biallelic frameshift mutations in the A10-coding mononucleotide microsatellite of the TβRII gene. TβRII displayed biallelic inactivation in the HCT116 CRC cell line. The reconstitution of TβRII signaling in HCT116 cells significantly decreased sialylation of cell surface proteins such as β-integrin without influencing β-integrin protein turnover (Lee et al., 2013), which suggests a relationship between sialylation and the classical mutational inactivation of TβRII in CRC cells (Table 1) (Lee et al., 2013; Lee et al., 2015; Ferreira et al., 2018).

*FUT3* and *FUT6* are involved in the synthesis of Lewis antigens, including the sialyl-Lewis^X^ (sLe^X^) and sialyl-Lewis^A^ (sLe^A^). Fucosylation of TβRI by *FUT3* and *FUT6* regulates the activation of the receptors (Fig. 3), leading to CRC cell migration and invasion by EMT (Hirakawa et al., 2014). In addition, highly expressed Lewis Y (Le^Y^) is observed in ovarian carcinoma-derived cancers. A detailed study in ovarian carcinoma-derived RMG-I cells showed that TβRI and TβRII had high levels of Le^Y^ structures which promoted the response of to the TGF-β-mediated phosphorylation of ERK, AKT and SMAD2/3 (Li et al., 2012). This finding indicates that the modification of TGF-β receptors with Le^Y^ is involved in the regulation of the TGF-β/SMAD pathway and in non-SMAD signaling.

### Effect of glycosylation on TGF-β co-receptor function

TGF-β signaling is initiated by the binding of TGF-β to TβRI and TβRII. In addition to these two classical signaling receptors, betaglycan, endoglin and neuropilins also regulate TGF-β signaling as co-receptors (Nickel et al., 2018). Both betaglycan and endoglin are highly glycosylated with *N*-linked and *O*-linked glycans, with one difference being that betaglycan has GAG chains that are not found on endoglin (Fig. 3, Table 1) (ten Dijke et al., 2008; Nickel et al., 2018). Betaglycan is a member of the dually modified transmembrane proteoglycan (DMTP) family, the members of which are composed of a core protein with covalently linked heparan sulfated (HS) and/or chondroitin sulfate (CS) GAG chains (Jenkins et al., 2018). Betaglycan is associated with the enhancement of TβRI/SMAD2/3 signaling (Lopez-Casillas et al., 1991; Esparza-Lopez et al., 2001). In contrast, endoglin is highly expressed on endothelial cells and inhibits TβRI/SMAD2/3 signaling while promoting activin receptyor-like kinase 1 (ALK1)/SMAD1/5 signaling (Lebrin et al., 2004). Glycosylation changes of betaglycan have been observed during signaling. In osteoblast-like cells, betaglycan binds to basic fibroblast growth factor (bFGF) through its heparan sulfate chains, while binding to TGF-β via its core protein. This study suggests that betaglycan might play a physiological role as a bifunctional growth factor-binding protein (Andres et al., 1992). The proper *N*-glycosylation of endoglin is crucial for directing it to exosomes (Meurer et al., 2019). Defective *N*-glycosylation of endoglin has been shown to interfere with its membrane localization (Lux et al., 2000). When liver cells were treated with tunicamycin to block the *N*-glycosylation of endoglin, aberrant trafficking of endoglin was observed.

Neuropilins (NRPs) constitute a family of transmembrane proteins that include NRP1 and NRP2, in which NRP1 undergoes *N*-linked glycosylation (Table 1) (Pellet-Many et al., 2008). Both of these neuropilins play roles as co-receptors in multiple cellular signaling cascades (Guo and Vander Kooi, 2015). NRP1 can capture and activate TGF-β by acting as a high-affinity co-receptor for both the latent and active forms of TGF-β1 (Glinka and Prud’homme, 2008; Glinka et al., 2011). In fibrotic livers and activated hepatic stellate cells (HSCs), galectin-1 (Gal-1) and its bound proteins could recognize the *N*-glycans on NRP1. This glycosylation-dependent Gal-1/NRP1 interaction activated the formation of the NRP1/TβRII complex and induced the TGF-β-like signaling pathway to promote HSC migration in the absence of TGF-β (Wu et al., 2017).

### Effect of glycosylation on SMAD protein function

SMAD2 is a crucial component of TGF-β intracellular signaling. A recently published study showed that SMAD2 can be glycosylated by *O*-GlcNAc and *O*-GalNAc glycans at the site of Ser110 in the MH1 domain in MCF7 breast cancer cell line (Table 1) (Gotoh et al., 2020). Mutation of Ser110 to alanine in SMAD2 attenuates of its translocation into the nucleus in response to TGF-β stimulation. The SMAD2 glycosylation is neither dependent on the C-terminal phosphorylation of SMAD2 nor affected by TGF-β1 treatment of the cells. Of note, when MCF7 cells were treated with 17β-estradiol for more than 6 hours, an inhibition of SMAD2 glycosylation was observed (Gotoh et al., 2020).

## Glycosylation changes in TGF-β-induced EMT

**Figure 4:**
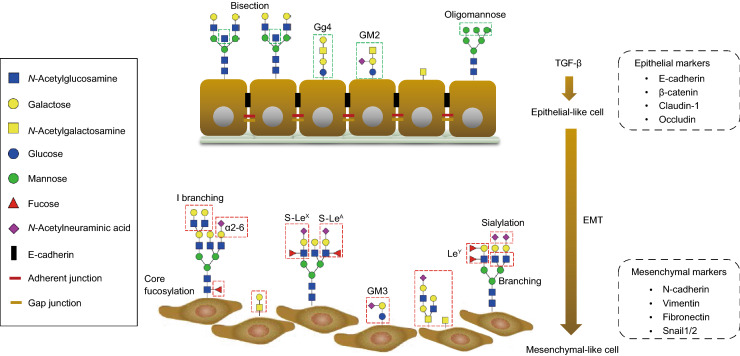
**Glycosylation changes in TGF-β-induced EMT**. During TGF-β-induced epithelial–mesenchymal transition (EMT), the epithelial cells lose their cell-cell contact and apical-basal polarity, acquiring a mesenchymal phenotype with enhanced motility and invasion ability. Upon EMT, epithelial markers including E-cadherin, β-catenin, claudin-1 and occludin are downregulated and mesenchymal markers such as N-cadherin, Vimentin, Fibronectin and Snail1/2 are increased. Glycosylation changes occur during EMT. Different types of changes are shown in the red-dashed boxes, highlighting changes in *O*-glycans and increased expression of branched, core fucosylated and sialylated *N*-glycans. In addition, the Lewis antigens (S-Le^X^, S-Le^A^ and Le^Y^) of *N*-glycans also upregulated within this process. The composition of the GSLs changed, as showed by the depletion of Gg4 or GM2 and expression of GM3 during TGF-β-induced EMT.

TGF-β-induced EMT is a key step for cancer cell invasion and metastasis and is accompanied by the aberrant expression of certain glycosyltransferases. The latter results in varying expression levels of glycolipids and cell-surface glycoproteins and contributes to the development of cancer (Lange et al., 2014). Analysis of the glycome and mRNA transcriptional profiles before and after stimulation of (normal and cancer) cells by TGF-β in several EMT models revealed upregulation or downregulation of specific glycan structures and glycogenes involved in biosynthesis of *N*-glycans, *O*-glycans and GSL-linked glycans (Fig. 4) (Li et al., 2016). The results from all these studies indicate the importance of the cellular glycosylation pattern in both the EMT process and the maintenance of the mesenchymal state.

### Role of *N*-glycans in TGF-β-induced EMT

*N*-glycosylation has been demonstrated to be involved in TGF-β-induced EMT, including branching, bisection, core fucosylation and sialylation (Fig. 4, Table 2). Consequently, the activity of *MGAT5* promotes TGF-β-induced EMT via the retention of TβRI/II at the cell surface (Partridge et al., 2004). Inhibition of *MGAT5* expression, which blocks the generation of branched *N*-glycans, profoundly suppressed TGF-β-induced EMT mediated by binding of galectin-3 to *MGAT5*-modified *N*-glycans in hepatocytes and prevented liver fibrosis. The target glycans are found on TGF-β receptors and delay ligand-induced TβRI/II internalization and further inhibit TGF-β signaling (Partridge et al., 2004). In the MKN45 gastric cell line in which *MGAT5* was overexpressed, there was an impairment of cell-cell interactions and reduced contact inhibition. *MGAT5*-knockout cells retained an epithelial morphology, as characterized by the high expression levels of E-cadherin (Pinho et al., 2009; Pinho et al., 2013). Conversely, *MGAT3* catalyzes the addition of bisecting GlcNAc and competes with *MGAT5*, resulting in an increased number of bisected structures and decreased branching. *MGAT3* overexpression inhibited TGF-β-induced cell motility and the EMT in a human breast cancer MCF10A cell line and the GE11 mouse cell line (Xu et al., 2012b). A further study reported that *MGAT3* induced a delay in the turnover rate of E-cadherin making it more stable on the cell membrane. The latter contributes to the formation of adherens junctions, thereby preventing clathrin-dependent E-cadherin endocytosis, and may play a role in tumor suppression (Pinho et al., 2009).

**Table 2:** Glycosylation changes during TGF-β induced EMT

Glycan type	Cell type	Altered glycan or glycan-related gene			Resulted phenotype	Reference
		Glycan	Gene	Promotion /inhibition		
*N*-glycan	Human breast cancer cell line, Mouse mammary epithelial cells	α2,6-sialic acid linked *N*-glycans ↑	*ST6GAL1* ↑	Promotion	Enhanced cell migration/invasion	(Lu et al., 2014)
	Non-tumorigenic mouse hepatocyte cells, Murine tumor cells	β1,6 branching *N*-glycans ↑	*MAGT5* ↑	Promotion	Enhanced cell migration/invasion	(Kamada et al., 2012; Xu et al., 2012b)
	Human breast cancer cell line, Mouse mammary epithelial cells	Bisecting *N*-glycans ↓	*MGAT3* ↓	Inhibition	Reduced cell motility	(Xu et al., 2012b)
	Human renal epithelial cells, Human giant lung carcinoma cells	Core fucosylation ↑	*FUT8* ↑	Promotion	Enhanced cell migration/invasion	(Lin et al., 2011)
					Development of an emphysema-like phenotype	(Wang et al., 2005)
*O*-glycan	Human breast cancer cell line		*GALNT14* ↑	Promotion	Enhanced cell migration/invasion	(Huanna et al., 2015)
	Human prostate epithelial cell line		*GALNT3* ↑ *GALNT6* ↑	Promotion		(Ding et al., 2012*)*
Glycosphingolipids	Human and mouse breast epithelial cell	GM2, Gg4 ↓	*β3GalT4* ↓	Inhibition	Reduced cell motility	(Guan et al., 2009)
	Human lens epithelial cells	GM3 ↑		Promotion	Enhanced cell migration	(Kim et al., 2013)
	Human mammary epithelial cells	GD2 ↓		Inhibition	Inhibited metastasis	(Sarkar et al., 2015)
Other structure motifs	Human colorectal cancer cell line	Sialyl-Lewis^A^ ↑	*FUT3* ↑	Promotion	Enhanced cell migration/invasion	(Hirakawa et al., 2014)
		Sialyl-Lewis^X^ ↑	*FUT6* ↑			
	Human ovarian cancer cell line	Lewis^Y^ ↑	*FUT4* ↑	Promotion	Enhanced cell migration/invasion	(Li et al., 2010)
	Human keratinocyte cells	Sialylated *N*-glycan ↑		Promotion	Enhanced cell migration/invasion	(Du et al., 2015)
	Human breast cancer cell line	I antigen ↑	*GCNT2* ↑	Promotion	Enhanced cell migration/invasion/lung metastasis	(Zhang et al., 2011)

Core fucosylation of *N*-glycans shows an essential role in activation of TGF-β signaling. In human renal proximal tubular epithelial cells, blocking the expression of *FUT8* for core fucosylation caused the inactivation of TGF-β/SMAD2/3 signaling and resulted in the attenuation of the EMT (Lin et al., 2011). Terminal α2,6-sialylation significantly increased during TGF-β-induced EMT in the GE11 murine epithelial cell line (Lu et al., 2014). This outcome was demonstrated by the increased expression of β-galactoside α2,6-sialyltransferase 1 (*ST6GAL1*) during TGF-β-induced EMT, which catalyzes the addition of terminal α2,6-sialic acid linkages on galactose (Fig. 4). Overexpression of *St6gal1* promoted the induction of the mesenchymal marker α-smooth muscle actin (α-SMA) and accelerated the EMT process. In contrast, knocking down *St6gal1* in the GE11 cell line inhibited the TGF-β-induced EMT and upregulated the epithelial marker E-cadherin. This effect was also observed in the MDA-MB-231 human breast cancer cells, and the mesenchymal phenotype of this cell line was partially reversed upon *ST6GAL1* knockdown, as determined by an increase in the epithelial marker E-cadherin and a decrease in mesenchymal markers, including α-SMA, β1 integrin and fibronectin (FN) (Lu et al., 2014).

### Role of *O*-glycans in TGF-β-induced EMT

Numerous studies indicate that structural changes in mucin type *O*-glycosylation could induce EMT and promote cancer cell invasiveness and metastasis (Gu et al., 2010; Mi et al., 2011; Lynch et al., 2012). Mucin-type *O*-glycosylation is catalyzed by enzymes in the *N*-acetylgalactosaminyltransferase (GALNT) family, including *GALNT14*. Clinical data have shown that *GALNT14* is highly expressed in various human cancers, such as breast cancer (Huanna et al., 2015) and hepatocellular carcinoma (Lin et al., 2014), and plays an important role in regulating malignant characteristics, as is exemplified by an increased expression of some mesenchymal markers N-cadherin and vimentin and TGF-β (Table 2) (Huanna et al., 2015). Mucin type *O*-glycosylation is also plays an important role in TGF-β-induced EMT in human prostate epithelial cell lines by regulating the reactivity of oncofetal fibronectin (onfFN) (Freire-de-Lima et al., 2011). In fetal cells and cancer tissues, there is a significant increase in onfFN upon treatment with TGF-β. The reactivity of onfFN requires the addition of an *O*-glycan at a specific Thr, catalyzed by *GALNT3*, and/or *GALNT6* (Freire-de-Lima et al., 2011; Ventura et al., 2018). When both *GALNT3* and *GALNT6* of onfFN are depleted from cells, the TGF-β-induced EMT process is blunted. Further investigation showed that only *O*-glycosylated onfFN, and not FN lacking *O*-GalNAc, can promote TGF-β-induced EMT (Table 2) (Ding et al., 2012). Although the molecular mechanism of this unusual glycan-modified FN-promoted EMT is unclear, this *O*-glycosylated onfFN might be a potential target for cancer therapy.

### Role of glycosphingolipids in TGF-β-induced EMT

The inhibition of GSLs in the TGF-β-induced EMT process has been reported in normal murine NMuMG mammary gland cells and human MCF7 mammary carcinoma cells. During the TGF-β-induced EMT process, the composition of the GSLs changed in these cell lines: in NMuMG cells, Gg4 or GM2 was depleted or decreased (Guan et al., 2009), and in HCV29 cells, GM2 was decreased (Fig. 4, Table 2) (Guan et al., 2009). The use of the GlcCer synthase inhibitor D-threo-1-(3’,4’-ethylenedioxy)-phenyl-2-palmitoylamino-3-pyrrolidino-1-propanol (EtDO-P4) to inhibit the synthesis of GSLs led to upregulated mesenchymal markers, including N-cadherin, vimentin and fibronectin, and promotion of cell motility. The enhanced EMT by GSL depletion or TGF-β-induced EMT can be abrogated by the addition of exogenous GM2 and Gg4. In addition, blocking the expression of GD3, which is a ganglioside involved in GD2 biosynthesis, initiates the EMT process, and the mesenchymal phenotype is maintained (Sarkar et al., 2015). Inhibition of another ganglioside, GM3, by the inhibitor *d*-*threo*-1-phenyl-2-decanoylamino-3-morpholino-1-propanol (*d*-PDMP) or by knocking it down led to mitigated cell motility and blocked TGF-β-induced EMT through a potential interaction with TβRs (Kim et al., 2013). In contrast, elevated levels of ganglioside GM3 positively regulates cell migration and TGF-β-induced EMT in lens epithelial cells.

### Role of other glycan epitopes/terminal structures in TGF-β-induced EMT

Sialic acids, a family of nine-carbon backbone monosaccharides, are usually overexpressed in cancer cells to protect malignant cells from the cytotoxic effect of natural killer cells (Chen and Varki, 2010; Chaudhary et al., 2019). Du et al. used a chemical reporter strategy and visualized the dynamic changes in sialylation during TGF-β-induced modulation of epithelial plasticity in human keratinocyte HaCaT cells. Using 3Fax-Neu5Ac, a global inhibitor of sialylation, the EMT process was promoted in the early stage, and once the cells entered the mesenchymal-like state, the effect was no longer significant (Du et al., 2015). Moreover, upregulation of I-branching β-1,6-*N*-acetylglucosaminyl transferase 2 (*GCNT2*) has been observed in TGF-β-induced EMT in basal-like breast tumors and were correlated with metastasis phenotypes (Table 2) (Zhang et al., 2011). This enzyme is a member of the β-1,6-*N*-acetylglucosaminyltransferase family and is involved in driving the progression of breast tumors and malignancies (Zhang et al., 2011). Overexpression of *GCNT2* promoted TGF-β-induced EMT, which was accompanied by enhanced breast cancer cell migration, invasion and lung metastasis (Andergassen et al., 2015). Knocking down *GCNT2* showed the opposite regulatory effect on these EMT-related cellular processes.

## Conclusion

In this review, we described evidence showing the role of specific *N*-glycans, *O*-glycans, and GSLs in TGF-β signaling and glycosylation changes during the TGF-β-induced EMT. Several studies have recently demonstrated that *N*-glycosylation of TβRII can regulate TGF-β signaling by remodeling TGF-β receptors and inhibiting endocytosis. The EMT process is accompanied by changes in glycosylation, such as an increase in sialylation and the number of sLe^X^ and sLe^A^ structures. However, in most cases, the molecular mechanisms and clinical significance of specific glycosylation changes during EMT are still unclear.

Many studies have contributed to the current knowledge of glycosylation of cells in TGF-β signaling. To determine the activity of glycosyltransferases and glycosidases *in vitro*, researchers have developed, and continue to improve, chromatographic, radiochemical or spectrophotometric techniques to follow the loss of substrates or the formation of the reaction products (Laughlin and Bertozzi, 2009; van Kooyk et al., 2013; Alteen et al., 2020). The lectin microarray (Zhang et al., 2016) and mass spectrometry (Couto et al., 2018; Gargano et al., 2020) are used to check glycosylation profiles and to discover new glycan structures. These data need to be integrated with genomics and proteomic profiling studies that determine the changes in expression and localization of glycosyltransferases and glycosidases and link them to biological responses. It will be further important that these studies are complemented with functional studies in which the effect of misexpression of specific genes encoding for glycan modifying enzymes and their substrates. Moreover, the effect of cellular responses upon treatment with pharmacological small molecule inhibitors of glycan modifying enzymes or (if possible) the addition of glycan substrates or products on cellular responses will be informative. The technological advances and holistic approach to identify and functionally investigate changes in glycosylation, will help in the identification of new glycan markers and create inroads for the development of better diagnosis and improved therapies for cancer patients.
